# A Potential Role of the Renin-Angiotensin-System for Disturbances of Respiratory Chemosensitivity in Acute Respiratory Distress Syndrome and Severe Acute Respiratory Syndrome

**DOI:** 10.3389/fphys.2020.588248

**Published:** 2021-01-20

**Authors:** Swen Hülsmann, Sepideh Khabbazzadeh, Konrad Meissner, Michael Quintel

**Affiliations:** ^1^Universitätsmedizin Göttingen, Klinik für Anästhesiologie, Georg-August-Universität, Göttingen, Germany; ^2^DONAUISAR Klinikum Deggendorf, Deggendorf, Germany

**Keywords:** acute lung damage, respiratory chemoreflexes, neuronal control of breathing, brainstem, homeostasis

## Abstract

Acute respiratory distress syndrome (ARDS) represents an acute diffuse inflammation of the lungs triggered by different causes, uniformly leading to a noncardiogenic pulmonary edema with inhomogeneous densities in lung X-ray and lung CT scan and acute hypoxemia. Edema formation results in “heavy” lungs, inducing loss of compliance and the need to spend more energy to “move” the lungs. Consequently, an ARDS patient, as long as the patient is breathing spontaneously, has an increased respiratory drive to ensure adequate oxygenation and CO_2_ removal. One would expect that, once the blood gases get back to “physiological” values, the respiratory drive would normalize and the breathing effort return to its initial status. However, in many ARDS patients, this is not the case; their respiratory drive appears to be upregulated and fully or at least partially detached from the blood gas status. Strikingly, similar alteration of the respiratory drive can be seen in patients suffering from SARS, especially SARS-Covid-19. We hypothesize that alterations of the renin-angiotensin-system (RAS) related to the pathophysiology of ARDS and SARS are involved in this dysregulation of chemosensitive control of breathing.

## Introduction

Per definition, acute respiratory distress syndrome (ARDS) is characterized by an inhomogeneously distributed, noncardiogenic pulmonary edema and acute hypoxemia. Its presence is still associated with a high mortality. ARDS is triggered by various stimuli, such as sepsis, major trauma, and pneumonia. The underlying pathophysiology involves activation of the immune system, pneumocyte injury, surfactant dysfunction, and coagulopathies. It markedly impairs adequate exchange and consecutively oxygenation and carbon dioxide removal (Balibrea and Arias-Diaz, 2003; Ranieri et al., 2012; Fanelli and Ranieri, 2015). Patients with ARDS may present with alterations of the breathing pattern, and its regulation might not directly correlate with the O_2_ or CO_2_ partial pressures measured in the arterial blood (Spinelli et al., 2020). Of note, despite normalizing arterial pO_2_ and pCO_2_ by mechanical ventilation and/or extracorporeal lung support, patients might still present with respiratory rates far higher than expected or needed (Crotti et al., 2017). These patients might require high doses of sedation or even muscle relaxants and controlled ventilation to prevent patient self-inflicted lung injury (P-SILI). Interestingly, in acute cases of COVID-19 pneumonia (SARS), similar observations were made. Despite normalization of the arterial blood gases, COVID-19 patients continued to show forced breathing patterns that might additionally harm the already virus-altered lungs (Cruces et al., 2020; de Vries et al., 2020; Li et al., 2020; Marini and Gattinoni, 2020; Smit et al., 2020).

In this hypothesis and theory paper, we discuss potential mechanisms that might disturb respiratory chemosensitivity in patients with ARDS or SARS.

## The Renin-Angiotensin-System in ARDS

**Figure 1:**
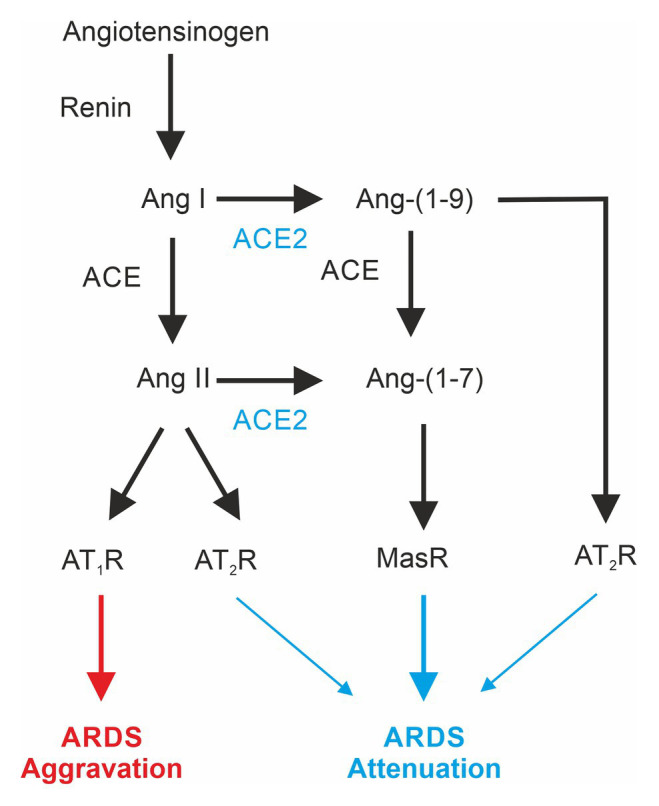
Differential effects of mediators of the renin-angiotensin system involved in acute respiratory distress syndrome (ARDS). ACE, angiotensin-converting enzyme; ACE2, angiotensin-converting enzyme 2; Ang, angiotensin; AT1-R, angiotensin II receptor type 1; AT2-R, angiotensin II receptor type 2; Mas-R, Mas-Receptor.

The renin-angiotensin-system (RAS; Figure 1) or renin-angiotensin-aldosterone system (RAAS) appears, apart from regulation of blood pressure, to be also involved in the pathogenesis of ARDS (Magalhães et al., 2019). Its main mediator, Angiotensin II (Ang II), is involved in inflammatory and fibrogenic processes in the lungs (Marshall et al., 2004; Hagiwara et al., 2009; Fletcher et al., 2017). Animal experiments in ARDS models demonstrate that the reduction of Ang II formation by inhibition of ACE exerts a protective effect (Imai et al., 2005, 2008; Shen et al., 2009). For example, the ACE inhibitor captopril is able to diminish oleic acid-induced severe acute lung injury in rats (He et al., 2007). Likewise, pharmacological inhibition or genetic deletion of AT1a receptors significantly mitigates lung injury (Raiden et al., 2002; Imai et al., 2005, 2008).

The angiotensin-converting enzyme 2 (ACE2; Donoghue et al., 2000; Tipnis et al., 2000), a homolog to the classical ACE, is also expressed in the lung (Hamming et al., 2004; Jia, 2016). The lack of ACE2 expression in ACE2-KO animals increases ARDS susceptibility, and moreover, inactivation of ACE in ACE2-deficient mice attenuates ARDS (Imai et al., 2005). ACE2 catalyzes the formation of angiotensin Ang-(1–7), which acts *via* the Mas-Receptor (Mas-R; Zambelli et al., 2012). Pharmacological activation of Mas-Rs or administration of recombinant ACE2 has been shown to exert lung-protective effects (Imai et al., 2005; Wosten-van Asperen et al., 2011). In addition, ACE activity is increased in ARDS-lungs, and ACE2 activity is reduced (Li et al., 2008; Wosten-van Asperen et al., 2011).

Taken together, these observations suggest that the ACE2-product Ang-(1–7) *via* the Mas-Receptor promotes protective effects in the lung, and shifting the RAS toward ACE/Ang II/AT_1_R has deleterious effects (Wang et al., 2019). Finally, ACE2 also cleaves Ang-(1–10) to angiotensin 1–9 acting *via* the AT_2_R, which has been shown to exert protective effects on ARDS development (Imai et al., 2005) and pulmonary hypertension (Cha et al., 2018).

Although in ARDS mice Ang II serum levels are elevated (Imai et al., 2005; Chen et al., 2013; Zou et al., 2014), data for humans are less clear. The *Ace* gene insertion/deletion (I/D) polymorphisms correlate with the susceptibility for and severity of ARDS (Marshall et al., 2002; Jerng et al., 2006; Adamzik et al., 2007; Tsantes et al., 2013) with those patients carrying a lower risk that are homozygous for the insertion (II) genotype (Adamzik et al., 2007). Since the ACE II genotype is associated with a lower serum ACE concentration (Rigat et al., 1990), one would expect lower ANG II serum levels. However, serum Ang II levels in humans are quite variable in ARDS as well as in control patients. Significantly higher Ang II serum levels in ARDS patients have never been reported (Wiberg-Jorgensen et al., 1983; Reddy et al., 2019). Nevertheless, a significantly higher Ang-(1–7) to Angiotensinogen [Ang-(1–10)] ratio as well as Ang-(1–9) to Ang-(1–10) ratio in ARDS survivors (Reddy et al., 2019) gives a hint of a protective effect of the ACE2. In addition, a pilot clinical trial using recombinant human angiotensin-converting enzyme 2 in ARDS revealed increased Ang-(1–7) levels but “did not result in improvement in physiological or clinical measures of ARDS in this small study” (Khan et al., 2017). Unfortunately, in this study, Ang-(1–9) levels were not tested.

## The Renin-Angiotensin-System in SARS

Coronavirus disease 2019 (COVID-19^1^) is a zoonotic disease caused by the novel SARS-CoV2 (Zhu et al., 2020). Although causing, in many cases, only mild symptoms, some patients develop a severe acute respiratory syndrome (SARS), which resembles ARDS in some but not all aspects (Gattinoni et al., 2020b,c; Marini and Gattinoni, 2020). The angiotensin-converting enzyme 2 is the receptor for SARS-CoV (Li et al., 2003) and SARS-CoV2 (Hoffmann et al., 2020).

In the initial phase of the COVID-19 pandemic, concerns about an increased risk for patients treated with ACE-inhibitors or angiotensin-receptor-blockers (ARBs) were raised (Kuster et al., 2020). Meanwhile, this topic has been studied extensively. In brief, no increase in the severity of COVID-19 or SARS-CoV2 infections have been found (Reynolds et al., 2020); in contrast, studies confirm a potential protective effect (Hippisley-Cox et al., 2020).

Interestingly, a considerable number of patients do not experience shortness of breath or dyspnea in the early phase of COVID-19 despite an already markedly impaired gas exchange, a status called silent hypoxia or silent or happy hypoxemia (Couzin-Frankel, 2020; Dhont et al., 2020; Ottestad et al., 2020). This phenomenon appears when lung compliance is still near normal but gas exchange is already impaired by ventilation/perfusion mismatch and functional shunt [non-ARDS type 1 (or type L); Gattinoni et al., 2020b]. SARS-CoV2 does not only infect the pulmonary epithelium, but heavily alters the vascular endothelium, causing impairment of its antithrombotic properties (McFadyen et al., 2020; Teuwen et al., 2020); thus micro-angiopathy and micro-embolisms can explain the alteration of the ventilation/perfusion ratio that is caused (Merrill et al., 2020). Moreover, pulmonary vasoplegia suspending partially or totally hypoxic pulmonary vasoconstriction leads to reasonable functional shunt (Chau et al., 2020).

However, these patients show mostly tachypnea (Chandra et al., 2020; Ottestad et al., 2020), clearly favoring the concept of an already increased respiratory drive and conflicting with the concept of a “failure to trigger the centrally mediated increase in respiratory rate” as put forward by Soliz (Soliz et al., 2020). The nearly normal compliance of the type L lung can explain the lack of dyspnea: As long as breathing efforts are not limited by the lungs’ elastance or external factors (Albashir, 2020). However, the increased respiratory drive can lead to severe hyperventilation with breathing efforts that create large negative pressure swings that lead to self-inflicted lung injury (P-SILI), thus promoting a shift to the H-type of COVID-19 pneumonia (Cruces et al., 2020; Gattinoni et al., 2020a; Smit et al., 2020).

Apart from this clinical alteration, it has been shown that plasma levels of angiotensin II of SARS-CoV2 infected patients were elevated (Liu et al., 2020; Wu et al., 2020), and moreover, plasma levels correlated to the viral load as well as to the degree of lung injury (Liu et al., 2020). An explanation for this is that the binding of SARS-CoV2 to virus-receptor ACE2 led to a downregulation of enzyme ACE2 in the lung tissue (Silhol et al., 2020), a mechanism that had been described already for SARS-CoV1 (Kuba et al., 2005).

## RAS and the Regulation Breathing

Ang II and Ang-(1–7) exert differential effects on the carotid body (CB) glomus cells. In CB glomus cells, Ang II increases the respiratory drive by activation of NADPH oxidase (NOX) and mitochondrial-mediated O_2_-production with the consequence that K^+^-channels are inhibited and voltage-gated Ca^2+^ channels are activated (Allen, 1998; Schultz, 2011). In contrast, Ang-(1–7) exerts an inhibitory influence on glomus cells *via* activation of nNOS and NO-mediated activation of K^+^ channels (Schultz, 2011; Fung, 2014). It is of note that chronic hypoxia upregulates the expression and function of AT1-receptors in the carotid body (Leung et al., 2000).

However, the stimulation of breathing by i.v. application of Ang II in dogs could not solely be attributed to alterations in the carotid body activity (Potter and McCloskey, 1979), thus suggesting a role of central chemosensory pathways. Injection of Ang II into the nucleus of the solitary tract (NTS), which relays the chemosensitive information from the CB, is able to increase the respiratory rate (Paton and Kasparov, 1999). Moreover, Ang II receptors are expressed on many neurons, including serotonergic neurons in the raphe nuclei (Allen et al., 1991), which contain central CO_2_-chemosensor neurons (Severson et al., 2003; Richerson, 2004; Bhandare et al., 2020). Although the mechanism of Ang II action in these neurons is not yet completely understood, it is known that Ang II regulates release and synthesis of serotonin in raphe neurons (Nahmod et al., 1978) and that Ang II decreases the resting K^+^ conductance in other types of brainstem neurons (Li and Guyenet, 1996).

ACE2 is also expressed in the mouse brainstem (Lin et al., 2008), particularly in raphe neurons (Doobay et al., 2007). The functional role of the Ang II or Ang-(1–7) in primary respiratory neurons of pre-Bötzinger Complex in the medulla has not been investigated yet, but solid evidence exists that Ang II or Ang-(1–7) modulate the activity of cardiac neurons neighboring the respiratory neurons in the ventral lateral medulla (de Moura et al., 2010) as well as neurons in the nucleus of the solitary tract (Diz et al., 2002). Several recent studies demonstrate that ACE2/Ang-(1–7)/MasR interacts in the CNS with different neurotransmitter systems, including GABA, dopamine, and norepinephrine (Gironacci et al., 2004; Stragier et al., 2005; Wang et al., 2016). MasR are robustly expressed in GABAergic neurons in the basolateral amygdala (BLA), and ACE2 overexpression increases the spontaneous postsynaptic inhibitory currents in this region (Wang et al., 2016).

## A Novel Hypothesis: Synthesis of the Obvious

**Figure 2:**
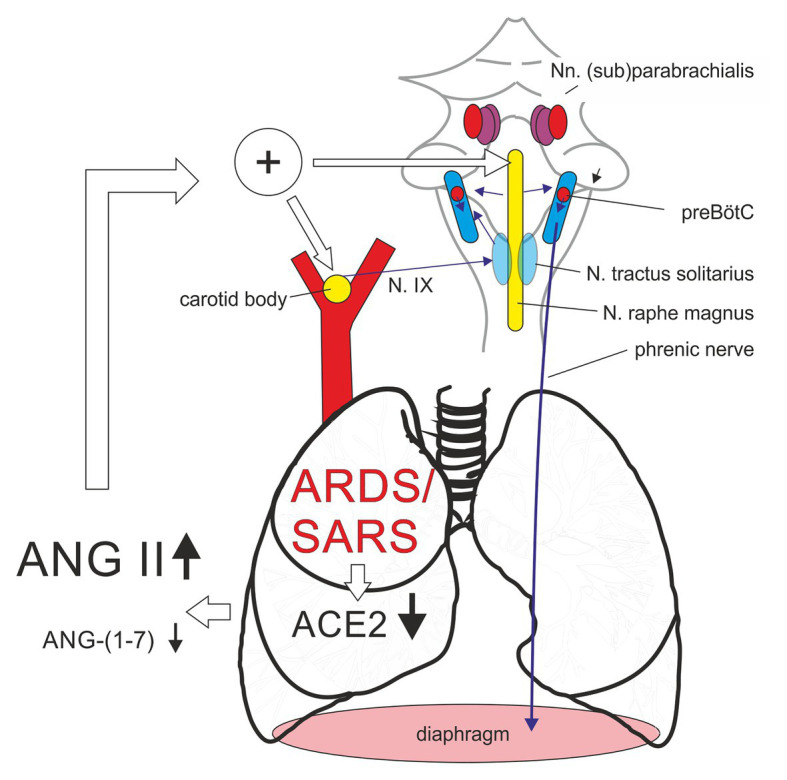
Alterations of renin-angiotensin-system (RAS) in the patient with acute respiratory distress syndrome or coronavirus-induced severe respiratory syndrome (SARS) led to an increase of respiratory chemosensitivity by an Angiotensin II (Ang II) mediated shift of the activity of the chemosensitive cell population in the carotid body and the medullary raphe. ACE2, angiotensin-converting enzyme 2; N. IX, glossopharyngeal nerve; preBötC, pre-Bötzinger complex.

Based on the literature reviewed above, we suggest the following hypothesis: In acute respiratory distress syndrome (ARDS) and in severe acute respiratory syndrome (SARS/COVID-19), alterations of the renin-angiotensin-system (RAS) signal a change of the chemosensitive reflex control of breathing, which results in an increase of the respiratory drive, which becomes independent from alterations of blood gases. Our hypothesis is based on the following key observation: In ARDS and especially in SARS/COVID-19, the RAS is dysregulated and shifted toward the ACE/Ang II/AT1R axis. This dysregulation is expected to stimulate, apart from any potential effect on the lung tissue, chemosensitive neurons in the brainstem and also chemosensitive cells in the carotid body (Figure 2), making them more sensitive to changes of CO_2_ and O_2_ and, thus, shifting their baseline activity and response curves to higher values.

## Discussion

Confirmation of this hypothesis requires a joint effort of clinical and basic scientists with broad knowledge in physiology and neurosciences. Experimental approaches should include *in vivo* and ex vivo studies in animal models of ARDS.

### What Types of Animal Models Are Available?

In general, so far, only animal models for the “classical” ARDS have been established and used, trying to mimic the uniform pathophysiology of this syndrome, characterized by a marked shunt volume and heavy, hard-to-move lungs. A COVID-19 affliction might – in the early phase – present with nearly normally compliant lungs but a heavily altered ventilation/perfusion (V/Q) ratio and a marked functional shunt volume, leading to severe hypoxia. The classical ARDS models have their clear limitations with regard to their transferability to clinical practice; they are what they are: models. To the best of our knowledge, a model for mimicking low V/Q and functional shunt does not exist and seems difficult to develop (Matute-Bello et al., 2008). Some of the “classical” ARDS models require intravenous application of agents, e.g., oleic acid (Schuster, 1994), and in others, the lung injury is induced by intratracheal application of the toxic agent, e.g., of acid (Imai et al., 2005) or bleomycin (Moore and Hogaboam, 2008). Data about alteration of respiratory control in animal models of acute lung injury and ARDS are limited. In the bleomycin model, alteration of the respiratory drive is described, which is independent of the impairment of oxygen exchange in the lung tissue (Jacono et al., 2006; Hsieh et al., 2020; Litvin et al., 2020). Alteration of Ang II serum levels have yet not been analyzed in the bleomycin model but are confirmed, among others, in the acid-instillation model (Imai et al., 2005; Chen et al., 2013; Zou et al., 2014).

Mouse models for COVID-19 that allow the analysis of breathing regulation are more complicated to develop, not only because the animal experiments are hindered by the need of laboratories with high biosafety levels, but because the spike proteins of SARS-CoV and SARS-CoV2 have a much lower binding affinity to the murine ACE2 than to its human homolog (Lutz et al., 2020). However, transgenic mice have been developed that express the human ACE2 (McCray et al., 2007; Bao et al., 2020; Sun et al., 2020). To our knowledge, no experiments on chemosensitivity have been performed in later mouse models yet.

### How to Test Change of Chemosensitivity in ARDS Models?

Based on this hypothesis, it will be necessary to determine how the shift of the RAS toward the ACE/Ang II/AT1R axis influences the target cell population of the chemosensitive reflex. Therefore, experiments in animal models of ARDS and SARS are necessary to establish the cellular basis of alteration of neuronal control of breathing. There is a wide range of experimental tools available that allow addressing chemosensitivity of the respiratory network at different levels. Experiments could be performed in acutely isolated brainstem slices, allowing measurement of the direct response of cells to alteration of CO_2_ or O_2_ (Gourine et al., 2010; Rajani et al., 2018).

Alteration of chemosensitivity in mice with ARDS can also be tested *in vivo* using whole body plethysmography, where alteration of tidal volume and respiratory rate can be analyzed in animals exposed to different levels of CO_2_ or and/or O_2_ (Bissonnette and Knopp, 2004; Hsieh et al., 2020). Moreover, the whole respiratory network can be analyzed in an arterially perfused preparation [the working heart brainstem preparation, WHBP (Paton, 1996; Dhingra et al., 2019)], which has the advantage that it allows testing for alterations of the chemosensitivity and respiratory drive that are independent from the injury of the lung since blood gas can be controlled *via* the perfusate.

### Alternative Mechanisms of Modulation of Respiratory Drive in ARDS

Ang II might increase respiratory drive *via* activation of carotid body (CB) glomus cells (Allen, 1998; Schultz, 2011, chemosensitive neurons of the raphe Severson et al., 2003 #3721; Richerson, 2004 #4146; Bhandare et al., 2020 #304), and in the relay nucleus of the solitary tract (NTS; Paton and Kasparov, 1999 #14118). However, further experiential effort is necessary to identify ARDS-dependent changes in other areas of the respiratory network, whether RAS may be involved directly or indirectly. This includes retrotrapezoid body (RTN) and the parafacial respiratory group, the pontine parabrachial/Kölliker-Fuse complex (pB/KF) as well as the ventrolateral medulla with BötC, preBötC, and VRG (Li and Guyenet, 1995).

Apart from its action on neurons, Ang II might be involved in alterations of astrocytes-dependent modulation of the respiratory network. Indeed, in many regions of the brain, AT-receptors have been found to be expressed on astrocytes (Sumners et al., 1991; Tallant et al., 1991; Gebke et al., 1998). Moreover, sequencing data indicate MasR-expression in astrocytes at least in older animals (Clarke et al., 2018). Whether the O_2_-sensitive astrocyte population in the medulla (Gourine and Funk, 2017; Rajani et al., 2018) or the population of CO_2_-sensitive astrocytes in the retrotrapezoid nucleus [RTN; (Gourine et al., 2010)] also expresses AT1R, AT2R, or MasR remains to be investigated.

From the beginning of the 1970s, it has been postulated that lung fibrosis can change breathing by alteration of lung reflexes (Guz and Widdicombe, 1970; Mansoor et al., 1997; Schelegle, 2003). Recently, lung reflex receptors, e.g., J-reflex, head deflation reflex, and Hering-Breuer inflation reflex, were again suggested to contribute to ARDS- and SARS-induced modulation of ventilatory response in patients (de Vries et al., 2020).

### Are There any Potential Secondary Effects of Elevated Angiotensin II?

Focus of the research should be extended beyond the direct effects of, e.g., Ang II on the target cells. Since Ang II is involved in the inflammatory response of the body, secondary neuroinflammatory effects that might modulate the neural control of breathing have to be considered as well (Pena-Ortega, 2019). Indeed, the elevated level of pro-inflammatory cytokines in critically ill COVID-19 patients sheds new light on this topic (Herold et al., 2020; Huang et al., 2020; Schett et al., 2020). Many of these mediators have also be found to be elevated in classical ARDS (Tzouvelekis et al., 2005), and their expression is often stimulated by Ang II (Han et al., 1999; Nakamura et al., 2002; Luther et al., 2006; Qi et al., 2011). For IL 6, IL-1β, and TNF-α, stimulatory effects in the carotid body have been demonstrated (Fan et al., 2009; Del Rio et al., 2012), and there is little doubt that these three cytokines can have potentially stimulating effects also on respiratory and chemosensitive neurons in the brainstem (Kawasaki et al., 2008; Pena-Ortega, 2019). Along with this, it has been recently shown that ARDS is associated with a specific modulation of the post-hypoxic frequency decline, a component of the respiratory chemoreflex (Hsieh et al., 2020). Further, it has been previously shown that carotid body chemosensitivity is upregulated even before the presence of severe lung injury pathology (Jacono et al., 2006). Similarly, 2nd-order NTS neurons have also been implicated in mediating a sensory-plasticity after lung injury (Getsy et al., 2019).

## Conclusion

In summary, imbalance of the renin-angiotensin-system in ARDS and SARS is expected to have substantial impact on the neuronal control of breathing and the chemosensitive reflex of the human body. While our hypothesis awaits experimental confirmation, it might lead to new therapeutic concepts and treatment options for intensive care patients with acute lung injury.

## Author Contributions

SH and MQ conceptualization, writing – review and editing. SK and KM writing – review and editing. All authors contributed to the article and approved the submitted version.

### Conflict of Interest

The authors declare that the research was conducted in the absence of any commercial or financial relationships that could be construed as a potential conflict of interest.

## Footnotes

^1^Novel Coronavirus (2019-nCoV). Situation Report – 22, WHO, February 12^th^, 2020, PDF downloaded July 28^th^ 2020.
